# A Collection of Memoirs on Military Medicine, Surgery and Pharmacy

**Published:** 1845-07

**Authors:** 


### Recueil de Mémoires de Medicine, de Chirurgie, et de Pharmacie Militaires, Vol. 57. Paris, 1844.

### A Collection of Memoirs on Military Medicine, Surgery and Pharmacy.

This new volume of Memoirs, issued under the superintendence of the French Council of Health of the Army, contains several interesting papers, abstracts of some of which we proceed to lay before our readers.

1. *Acute Dysentery with Detachment of the Mucous Membrane of the Large Intestine.*—Two cases are related by M. Catteloup, an attaché of one of the hospitals in Algeria. The *first* occurred in the person of a chasseur, aged 24, who had always enjoyed robust health, and was of sober habits. He was admitted into the hospital July 2, for a diarrhœa which had lasted several days, and was then accompanied with bloody stools. His affection, at first slight, soon became very grave, the bloody stools augmenting in number daily, and being accompanied with great pain and a quick full pulse. His treatment was antiphlogistic, and consisted in cupping the hypogastric region, bleeding from the arm and the application of leeches to the anus. Starch injections, cataplasms to the abdomen, and an abstinent diet were also employed, and three of the following pills, well known in French practice as Segond's pills, given at night. Ipecac. gr. j., Calomel gr. $\frac{1}{2}$, Opium gr. $\frac{1}{8}$. However, the symptoms progressed from worse to worse, and he died on the 13th. At the autopsy the small intestine was found to be healthy, but the large offered every lesion seen in dysentery. From the cæcum to the rectum it was covered with sanious or puriform discharge, upon the removal of which, engorgement, ramollissement, ulcers of variable size, and even gangrenous eschars were found. On opening the sigmoid flexure a cylindrical membrane, 32 centimetres in length, was observed. It so much resembled a portion of small intestine as at first to be mistaken for it. It was yet adherent at some points to the inner surface of the canal, and was believed to be a false membrane until its removal exhibited the muscular coat of the intestinal canal very distinctly. In the *second* case, the patient was a strong, healthy, but dissipated man, æt. 30, who, after suffering from intermittent diarrhœa for some weeks, was exposed to great changes of temperature and severe labour in a long march. The symptoms were soon converted into those of dysentery, and then became complicated with peritonitis. After death a perforation of the transverse colon and consequent abscess was found; the mucous membrane of the small intestines was quite healthy, but that of the large more or less diseased in its whole extent, presenting in different points ulcers and eschars. Near the perforation, a detachment of the mucous membrane, 35 centimetres in length, was observed, but which still retained some adhesions. An abscess was discovered in the substance of the anterior border of the liver.

The preparations in both these cases were submitted to a careful anatomical and microscopic examination by the professors of the Val-de-Grace, the result of which was, that no doubt whatever existed in their minds as to these being detached portions of the mucous membrane. The preparations are preserved, and are, the editor of the volume observes, of great importance, as being the only authentic examples of a detachment of a large portion of the mucous membrane from its mucous coat. In fact, the existence of such a lesion has been denied by most eminent pathologists, who have regarded such producticns as always pseudo-membranous.

2. *Acute Pulmonary Abscess.*—Subsequent pathological research has confirmed Laennec's statements of the rarity of the termination of pneumonia in abscess. M. Laveran relates the particulars of three cases occurring in soldiers. He observes that, being well aware how frequently the abscess resulting from softened tubercle, and even pus in dilated bronchi, have been mistaken for acute abscess, he has conducted his investigations with proportionate care. So rare indeed are genuine examples of these cases, that M. Grisolle has only been enabled, in his important treatise on pneumonia, to collect 22 of these. The author believes that the opinions of Dupuytren upon the nature of the different forms of cellular tissue, and their corresponding lesions, may be adduced with advantage in explanation of the rarity of these cases. That observer admitted three forms, the serous, adipose, and fibrous, œdema and abscess being the lesion of the first-named, gangrene of the adipose, and abscess with strangulation of the last. Thus we see why, in erysipelas, abscess is so common in the eyelids and so rare in the external ear. Does not the fibrous nature of the pulmonary structure explain the rarity of abscess?

The three cases detailed were examples of pleuro-pneumonia, in two of which it occurred on the left, and in one on the right side, and as the two former were also complicated with pericarditis, they confirm, as far as they go, the opinion long entertained at the Val de Grace, of the greater frequency of pericarditis in left than in right pleuro-pneumonia. The abscesses in these cases occupied the inferior lobes. The diagnosis of the abscess in each case was much obscured by the pleuritic effusion. In one case the gargouillement that was heard was naturally suspected to arise from a far more frequent cause, tubercular cavity. In the first case the expectoration was loaded with blood, as in one reported by Martin Solon. In the two other cases, with all the local signs of pleuro-pneumonia, there was the remittent fever so characteristic of suppuration. In reference to the effect of the pericarditis upon the pneumonia, M. Laveran observes.

"In theorizing upon the subject, the doctrine of irritation furnishes the following explanation. Bearing in mind that when the irritation of the pulmonary substance is permanent, as in the case of tubercle and foreign bodies, abscess is the rule and not the exception, we inquire whether the co-existence of the pericarditis has not proved a permanent cause, which, inducing an inflammation in the parenchyma, has caused it to proceed on to suppuration, just as foreign bodies in the intestines provoke phlegmonous inflammations of the iliac fossa or abscess at the margin of the anus, and as renal calculi induce lumbar abscess."

3. *Cases of Irritation produced by Hairs acting as Foreign Bodies.*—M. Judas, after adverting to recorded cases of obstinate inflammation of the eye, resulting from the undue prolongation of the delicate hairs of the caruncula, or the inversion of those of the eyelid, refers to two cases in which malingerers employed hair as a means of exciting inflammatory action. In both cases long needles, followed by bundles of hair, were introduced beneath the skin at the side of the patella, the hairs being then cut off level with the skin. In both instances very violent inflammation of the knee-joint resulted, followed by suppuration, and requiring the most active antiphlogistic measures. Two Italian practitioners have related each a case in which a pig's bristle became a source of great irritation. In one of these, a man, who had remained standing during part of an evening, experienced a slight pain upon the instep. This increasing next day, he found the large extremity of a bristle projecting. It was drawn out in part that, and in part the next, day, its length measuring 120 millimetres. Suppuration succeeded, and kept the patient lame for some days. The other case was similar, except that the bristle withdrawn was shorter, resembling exactly those of the brush which was employed to clean the shoes. Its removal was not followed by inflammation. A discussion arose between the reporters of these cases as to whether these substances had introduced themselves from without inwards, (for no suspicion was entertained of their having been purposely introduced,) or whether they had been accidentally swallowed with the food and eliminated by the skin!

M. Judas adds another case to these, which fell under his own observation. During a march from Metz to Paris, a captain of grenadiers complained to him of an intense pain at the sole of the foot, which compelled him to walk very lame. It was not until after a very attentive examination that a little thickening of the skin was seen at the point where the pain was complained of, on incising which the large extremity of a hair presented itself. This was soon removed, and all suffering ceased. It was about 25 millimetres in length, and exactly resembled the hairs of the leg. One of these doubtless had become detached, and slipping down to the sole, had been forced into a pore, dilated by the sweating of the foot.

" It is however a practical conclusion I wish to draw from this case. If it had occurred to a common soldier, one whom I might have suspected of simulation, might I not, in the absence of all external signs, have given course to this suspicion, and unwillingly have abandoned the unfortunate being to this source of pain, which would have tormented him much more than the officer, by reason of the weight of his equipments. Might not this cause, apparently so slight, have even given rise to tetanus in a hot country like Algeria?"

4. *Practical Observations upon Affections of the Anus.*—We may extract a few of the observations furnished by M. Barthelemy, who has paid much attention to this class of cases.

" *Fissure of the Anus.*—For some time past the therapeutics of this disease have singularly occupied the attention of surgeons. M. Trousseau declares it can be cured by injection of a strong decoction of *rhatany*, while others attribute a like efficacy to *ung. monesiæ.* Some surgeons apply caustic indiscriminately, others employ incisions. Like all other diseases, this presents differences as respects its cause and seat, and therefore it is plain that no one of these means will apply to all cases. Thus, in reference to causes, it is observable that syphilitic fissure, always infinitely less painful than ordinary fissure, disappears under the ordinary anti-venereal treatment, and by the use of opiated ointments during the acute stage, and of belladonnized mercurial ointment, or powdered calomel, when the inflammation is somewhat assuaged. In regard to the seat, it is well known that simple fissures, resulting from straining at stool, and situated in the middle of the rayed pleats of the anus, or a little higher up between the sphincters, have yielded to rhatany, monœsia, or nitrate of silver ointment, or, better still, to slight cauterization practised with this latter substance. But when the fissures are deep, and especially when they are situated very high between the sphincters, these secondary means generally fail, and it is only by complete and frequent cauterization, or better by incision, that the patient is relieved."

The author prefers in these cases incision, as less painful and more certain; but, where the patient will not consent to it, he applies the Vienna caustic freely to the fissured part, the nitrate of silver acting too superficially. When an operation is consented to, he makes an incision from 1½ to 2 lines in depth, along the whole length of the fissure, so as to convert it into a fresh wound, and declares that great success has attended this mode of procedure. He believes the spasm of the sphincters, which Boyer lays so much stress upon, is but consequent upon the irritation produced by the fissure, and that the division of the sphincter, whether by Boyer's method, or by the subcutaneous section as employed by Blandin, is unnecessary, and although great immediate relief results from it, relapse usually occurs. He objects, too, to M. Jobert's plan of excising the fissured part, as inflicting unnecessary pain, and leading to troublesome anal contraction.

" *Hæmorrhoidal Tumors.*—When these tumours have acquired a development, so that defæcation becomes very painful or even impossible, it is to surgery we must have recourse for the relief of the patient. Before doing this, relief, nay, even a cure, may sometimes be obtained by the employment of a well-combined plan of treatment, at the head of which may be placed bleeding from the arm, which will require repetition. I should like to add to this a little hydrotherapia, that is, I would advise cold water as the only drink, employing lavements and sitting-baths of the same. But when the tumours are old and pathologically organized, it is surgery which can alone avail. Until recently it was by the ligature, when their base was pediculated, and by excision, when it was large, that these tumours have been attacked. Now, however, strange to say, these means are almost abandoned, and the boldest operators recoil before the fear of hæmorrhage, which may kill immediately, and phlebitis, which may bring on, a little later, an equally fatal result. Cauterization is now the mode employed, some using concentrated acids, and others, the larger number, solidified Vienna caustic."

The author employs this latter substance formed into large sticks, in hæmorrhoids with a large base; but where he can find patients courageous enough, he much prefers the *actual cautery heated to whiteness,* which effects a more complete and rapid destruction of the tumour, and leaves a solid cicatrix which prevents a relapse. He relates a case in which a cure followed this treatment, which was first suggested to him by a paper by M. Begin in the *Annales de la Chirurgie.*

5. *Synovial Ganglions.*—M. Barthelemy has also a communication upon his mode of operating upon these bodies, when their dispersion is not effected by other means.

" Having raised a fold of skin at about an inch from the tumour, plunge into it the blade of a knife, strongly attached to a thin rounded handle, and considerably curved near its insertion. Pass this instrument, with its flat side downwards, to the cyst, which is to be divided in two, by passing through it horizontally from side to side, just as one cuts an almond. Draw out the knife by the same aperture it entered, following it in its retreat with the thumb of the left hand, which by proper pressure drives before it the contents of the cyst, at the same time that it opposes the admission of air; and then immediately establish compression by means of a plate of lead, which is to be continued for at least a fortnight."

This mode was proposed by M. B. in 1838, since when he and others have repeatedly performed the operation with success, when all other means have failed of giving relief.

In another part of this volume a case is related of a ganglion, occurring in the person of a young soldier. It was about the size of a pigeon's egg, and placed at the back of the left wrist-joint, just above the annular ligament. Breaking the walls of the cyst by forcible compression, evacuation of its contents with subsequent pressure, were tried in vain, and it was at length extirpated with little difficulty.

6. *On the Cure of Panaris by Mercurial Ointment.*—This is an account by M. Martin of a curious epidemic of whitlow, which affected many soldiers of a French Infantry regiment, while stationed in the Basque Provinces on the Spanish Frontier in 1835. The whole number affected amounted to 101 in 16 months, and 10 cases are related as examples. The inhabitants of these regions are remarkably robust, possess great corporeal agility, breathe a pure air, and partake of abundant food, containing a too large proportion of spice. It results that they enjoy a great exemption from internal maladies, and when these do occur, they are soon cured. But, at the same time, they are very liable to peripheral affection, such as erysipelas, dartres, phlegmons, furuncles, anthrax, hæmorrhoids, &c. After the regiment had arrived in this locality, and the soldiers had enjoyed a mode of life so different to that they had led in barracks, their health became better than it had been for eight years before. But this improved regimen, although it seemed to secure them against severe ailments, and impart to them much additional bodily activity, as the warm weather approached rendered them liable to a great variety of cutaneous diseases, especially the inflammatory affection of the hands, termed panaris. The indulgence in spirituous liquors seemed to have had much to do with this ; for the officers, who followed a temperate regimen, never were the subjects of the affection, and the soldiers, who did not indulge in excess, were also exempt, although exposed to the other causes, as immoderate use of spices, too violent exercise, and high temperature. The Spanish Basques, exposed to the same hygienic influences, but of much more sober habits than the French Basques, suffered much less frequently. External irritation, such as friction, contusions, &c., did not seem to have more than its ordinary influence in inducing this affection.

The disease was a serious one, being a most intense local phlegmasia, with corresponding constitutional derangement and local consequences. Suppuration was the mildest termination; for as the tendinous sheaths sometimes became implicated, caries of the phalanges and loss of a finger, was not an uncommon occurrence.

The treatment consisted in general and local bleeding, revulsives, narcotics, emollients, incisions, &c.; but, however carefully any of these means was employed, no arrest of the progress of the affection took place. The reporter having met in a journal with an account of the utility of the *mercurial ointment* in similar cases, gave it a trial with the happiest results ; for, rebellious as the disease had before shewn itself, it now became quite manageable. The part affected was rubbed with the ointment every alternate five minutes for two hours night and morning, a cataplasm being afterwards applied. Relief was so prompt and complete, that it was naturally believed the character of the disease had changed; but some cases happening to occur which were treated by the ordinary means displayed all the former virulence. Prior to the mercurial treatment resolution never occurred, and many most unfortunate terminations were observed: but, subsequently, the very reverse took place. After the troops left the locality and returned to their old quarters the disease never re-appeared.

7. *Cases of Scrofulous Caries.*—M. Chambolle, Surgeon to the Military Hospital of St. Denis, reports that scrofulous caries has shewn itself with remarkable frequency in his wards. In one year 18 cases were admitted, of which 12 died, 5 were discharged unfit for service, and 1 only remained convalescent. In nearly all the cases there had been exposure to cold and damp, and young soldiers were especially the victims of the disease. Its progress was sometimes very rapid, and at others very slow, the patients seeming to suffer little or nothing as regards pain, but becoming enfeebled by diarrhœa and fever. Treatment, whether active or temporizing, seemed of little avail, and whenever a patient presented himself with an abscess near a bone, some prolongation of his days was usually all that could be achieved. Antiphlogistics, iodine, tonics, caustics and the actual cautery were all tried in vain. Many patients suffered so little pain that they refused to submit to the severer caustics. Amputation and resection of the diseased bones were sometimes had recourse to with no better success than other measures. Several bones were frequently simultaneously affected, but the sternum, vertebræ, and ribs were those especially attacked.

8. *Fracture of the Leg, the patient walking and riding during twelve days after.*—An adjutant of dragoons, in jumping from a height, came flat upon his feet, and felt a severe pain in the middle of the leg, which obliged him to remain quiet for a few minutes. He soon, however, walked some miles, and for several days after continued to perform the duties of his post, which from the 9th to the 13th September, were of a peculiarly fatiguing character. During this period there were some pain and swelling, but on the last-named day, while descending a declivity, a bone was heard to snap, and he would have fallen to the ground but for the arm of a friend. Both bones were found fractured towards the lower part of the middle-third, the tibia being broken very obliquely. The case was then treated in the usual method, and the patient recovered the perfect use of the limb.

9. *Extirpation of the Eye in consequence of a gun-shot wound.*—A soldier was brought to the hospital 5th Dec. The ball had penetrated the right branch of the lower jaw and upper maxillary bones, and could nowhere be found. Notwithstanding prompt and judicious treatment, great cerebral disturbance came on, which soon became almost complete coma. From the first, the patient had always complained of intense pain in, and loss of the vision of, the left eye. This organ, at first slightly ecchymosed, eventually became highly inflamed, chemosed, swollen, and greatly projected from the socket. Its extirpation was effected with some difficulty, in consequence of the size of the organ, which was only sufficiently diminished by evacuating its fluid contents. At the bottom of the orbit the lost bullet was found and removed. The wound was brought together at its edges "without first filling it up with charpie, as most authors recommend, and so increase the violence of the inflammation." A wet compress was applied, and the patient, although the coma did not at once disappear, recovered perfectly.

10. *Preservation and Management of Leeches.*—The vast demand for leeches in France renders the sources of their supply a matter of national importance, and this memoir, prepared from numerous communications forwarded to the Council of Health, treats at great length of the best mode of forming ponds for them, and of preserving them in a healthy condition. Any of our leech-merchants who may feel disposed to try the experiment of constructing ponds for the sojourn and multiplication of these useful animals, instead of importing them, will find this paper a very useful one. We can make room only for an extract or two.

" *Re-application of Leeches.*—The most exact experiments have shewn that leeches which have sucked, if properly disgorged, not only recover their vigour and agility, and are fit for renewed use, but that this re-application may be made without the slightest danger. Different modes of disgorging them have been employed. Praised for a time, they have been abandoned as offering none of the expected advantages. The use of salt, nitre, tobacco-water, weak vinegar, alum, &c. is hurtful rather than useful. Under the influence of these irritating substances the leeches become restless and their muscles contract; but afterwards they languish and mostly die. Ipecac. recommended by Mr. Dick, has proved of no avail in the hands of others; while charcoal is of no more use than simple water. Some persons practise a deep incision along the posterior part of the body. This is a difficult process and kills so many that it should be abandoned.

" The only modes of disgorgement which careful experiment sanctions are *prolonged repose* and *pressure.* For the first, the leeches should be several times washed in river or spring water of a temperature of 60° or 70°, and then placed in earthen jars, at the bottom of which some clay has been laid. Those which are sufficiently strong will penetrate into the clay where they will remain, and all those who die should be at once removed. The water of the jars requires renewal every forty-eight hours for a fortnight. The survivors should then be put into large tubs of water containing some clay and aquatic herbs, and which are to be freely exposed to the air. Where there are leech-ponds they may be placed in them, when they will immediately penetrate into the mud lining the bottom. In five or six months they are fit for use.

" If, however, the leeches are urgently required, we must disgorge them by pressure. Shortly after the first application they are to be placed in water at about 70° to relax their tissues somewhat. After drying, they must be taken one by one in the thumb and fingers of the left hand covered with a napkin, while the thumb and fingers of the right hand, slightly moistened, are applied to the body of the leech so as to compress it from behind forwards several times until the blood has been forced out. If the leech is pressed violently, the elasticity of the muscles is destroyed, and the animal loses the power of contracting and dies. Too much blood forced out at once would also rupture the orifice. As they are disgorged the leeches should be thrown into vessels containing water at 70° to favour the complete evacuation of the blood. In a quarter of an hour this may be replaced by water at 50°, which requires renewal from time to time. Leeches so treated may be applied immediately after disgorgement, but this mode, useful in case of urgency, cannot be repeated often without injuring them."

We have the following observations upon the *Diseases of the Leech.*

" When in a state of liberty, the leech, in the enjoyment of the air, water, and light which it possesses in its natural pond, attached to the aquatic plants, or plunged in the slime, preserves all the characters of health in its form, colour, and vivacity. Any affection which would diminish its activity, or impede its power of penetrating into the mud, would soon deliver it into the hands of active enemies. When shut up in receptacles leeches are subject to several remarkable changes. If sometimes they preserve their health during several months, at others they become exhausted by an abundant exsudation, while the atmospheric vicissitudes to which they are subjected are found to be very hurtful to them. Sooner or later their form or colour change, they languish, and often succumb to various diseases in great numbers in a short time. It is difficult to obtain an exact account of these morbid effects. It has been said that the atmospheric electricity produces an important influence upon the mucosities they secrete. A cause of death sometimes arises in the putrefaction amidst which they are left, or the vitiation of the air by the decomposition of animal matters, yet with every precaution the mortality is frequently very great.

"M. Brossat first accurately described the diseases which are sometimes so promptly fatal to the leech. He observed that it was very difficult to preserve leeches collected before September, without seeing them attacked by three diseases, from which they are exempt only in winter. These he describes as—1. *La metallique*, a denomination given the leech on account of the change of *facies* it undergoes. Nodosities, in the shape of chaplets, form in various parts of their bodies. This very fatal disease may attack them from March to the end of May. It lasts about eleven days, during which they should not be used. They are to be kept in porous vessels formed of charcoal and burnt clay, which are suspended in a current of air, so as to keep the temperature of the water lower than that of the atmosphere. A little milk should be added to the water. 2. *Mucus*, which renders the leeches elastic and mucilaginous. This disease kills them by hundreds, lasting three days, and appearing from June to the end of August. A tepid bath should be used daily, the leeches being kept in the intervals in a mixture of water and powdered charcoal, to which a little honey has been added. 3. *Jaundice*. In this, the most fatal of all diseases, the leeches become as yellow as saffron, and resemble the stigmata of that plant. If not promptly succoured all perish at once. A small quantity of yellow fluid must be discharged from the tail of the animal by means of a needle. It is then washed in tepid water, and placed in water in which a little caramel has been dissolved.

"To these affections mentioned by Brossat, may be added one not less dangerous reported by M. Claude, viz. an inflammation of the intestinal canal. The leech increases in size, is surrounded by unequal rings, and its lips are pursed and bloody. In the digestive tube is found a fluid matter mixed with corrugated blood: the muscles are soft and soon putrefy. This disease M. C. considers contagious, and that it is produced by a decomposition of the mucosities secreted by the leech, when the changing of the water has been neglected. The leeches must be placed in large tubs with clay and aquatic herbs, or in pits dug for the purpose."

The volume terminates with an address delivered by M. Alquié, Chief Professor at the Val de Grace, upon the distribution of the prizes to the successful students. One or two passages from this we think are well worthy of being laid before our readers.

After observing upon the great advantages derivable from a modified reception of the *physiological doctrine*, almost repudiated by so many at the present day, he proceeds:

"Do not let the energy of my language surprise you gentlemen. You cannot measure as we can all the injustice, all the ingratitude of the present generation towards the genius of Broussais, in its complete forgetfulness of the benefits which he has multiplied in all branches of the practice of our art. You have not seen, as we have, these wards crowded with fever patients who were gorged with substantial *bouillons*, bark, and potions, to which they joined serpentary with musk, camphor, ether, and the whole tribe of incendiary stimuli. You have not seen inflamed wounds moistened with hot wine, and dressed with the most irritating ointments. You have not had to recoil with disgust at those large assemblages of venereal patients, saturated with mercury while gnawing chancres devoured their tissues, while large ulcers were surrounded by the worst forms of inflammation. This horrid ptyalism and hospital infection acquired a right of occupancy in our pox-wards, and the most lamentable circumstances were multiplying infinitely. Well, gentlemen, it is physiological medicine which has changed the face of all this, in teaching practitioners first to combat the inflammatory condition of the tissues, and thus remove from the disease one of its most important elements. At present the therapeutics of all these affections is much simplified. Physicians and patients benefit by this, without inquiring too minutely, or rather without inquiring at all, concerning the doctrines of the great master to whom they owe so much. It is just as in our social state we enjoy the immense prerogatives of a wide and wise liberty, without recollecting all the efforts and sacrifices it has cost our fathers to conquer it.

"Do we then, gentlemen, in the prevalent doctrines of the day find principles better established? Do the few facts upon which each is based suffice to efface all our medical creeds? Is it *Chemistry* which is to furnish the light which is to guide us all? No one views with more interest than myself the labours undertaken and pursued in common by physicians and chemists; but it must be acknowledged that, putting aside the elucidation of three or four diseases (diabetes, chlorosis, albuminuria, and gravel), these labours have not furnished medicine with anything but some additional sterile theories. Assuredly I do not forget the services which chemistry daily renders to the art of healing. She enlightens the physiology of the healthy man, and transporting her apparatus to the bedside of the patient, analyses the secretions and elucidates the nature of organic transformations. In teaching us the properties of bodies, and, by analogy, their influence upon the living economy, she spares us many trials and experiments which would not always be unattended with danger. She renders us eminent services in asphyxia, poisoning, and the correction of miasmata; and, thanks to her light, therapeutics is no longer an imperfect arsenal whence the physician could only take treacherous arms, neither the power or effects of which he could appreciate.

"But, in acknowledging the immense importance of chemistry, and in expressing the belief that it is yet from her that the science of man in health and disease has most to expect, we must still guard against her rash encroachments. The lessons of the past ought not to be lost to us, and the remembrance of the mischiefs caused to medicine by the chemists and iatro-chemists of all epochs, should render us very reserved when new pretensions are advanced. There would not be more wisdom in allowing ourselves to be now drawn into the exclusive consideration of the phenomena furnished by the organic fluids than there was, a while since, in attaching ourselves to an exclusive solidism. Do not let us forget that, in the vogue of some experiments, in the involuntarily exaggerated importance of some results, we must attribute a large part to that spirit of reaction which, in science as in politics, is one of the most deplorable tendencies of the human mind. Besides, gentlemen, make a conscientious inventory of all these wonders which they exalt with so great a noise of experiments and analyses and tell us if there is (as regards medicine) that which can constitute a doctrine in the place of that which has comprehended the labours of nearly an age, and which has only been produced by the combined and progressive efforts of Haller, Chaussier, Pinel, Bichat and Broussais.

"Does the *Numerical Method* satisfy us better. We cannot allow that it does. A simple process of verification *a posteriori*, never can be elevated to the dignity of a system, since it will be eternally true in medicine, that the problem is individual. Doubtless calculation may teach us in a general manner, in what proportions different diseases show themselves in this or that country, among men or women, the young or aged; in what proportion they exist in individuals of certain professions; but it can never establish from these enregistered facts that a certain formula of treatment must be applied to a certain order of facts, because nothing is so variable as the facts in medicine, and nothing is more rare than their perfect identity. We acknowledge that figures are a necessary base for the solution of a crowd of problems referring to the etiology and prophylaxis of morbid affections; and in an especial manner do statistics render ample services to military hygiene. They in some degree assist us in classing garrisons according to their degree of mortality, in ascertaining the prevalence of disease in different localities, the proportion of patients according to age, professions, duration of service, &c. &c. We conceive that, after the constant return of indentical observations, during a great number of years, the chief administration must take measures for the modification for the better of the various unfavourable conditions. It is a most laudable undertaking thus to assemble all the necessary materials, providing these materials be collected with a conscientious intelligence. This is the hygiene of administration enlightened by medicine; it is the accomplishment of duties devolving upon governments, guardians interested in the public welfare. But to desire, by means of figures, to establish a method of treatment, to which all cases belonging to a category of given affections must be submitted!—to say, *e. g.* that after copious bleeding, employed in 100 patients attacked with pneumonia, there have resulted 90 cures and 10 deaths; while the same number of patients, otherwise treated, have furnished 20 deaths and 80 cures —to conclude from these facts that since the first method had been followed with more success than the second, that it is *the true method,* the only *one that must be employed*—that it is to be constituted as a rule—to be elevated to the power of a law—this is what we cannot admit, and what experience and reason daily reject.

"In fact, may it not, does it not, happen that a new series of a hundred patients, attacked with pneumonia, may be composed of cases like the 10 which the first method has not been able to cure, and like those which have been cured by the second? and how then, we may ask, is an inflexible formula to be applied? Now, gentlemen, what we have here supposed, last winter you saw occur in our wards. We had frequently to direct your attention to the point, that the pneumoniæ and pleuro-pneumoniæ of the month of December and first half of January, yielded like enchantment to a bold antiphlogistic treatment, while those of February and March resisted it, and exacted, with greater reserve in blood-letting, the intervention of a different order of means. And nevertheless the patients of Feb. and March were affected with pneumoniæ like those of Dec. and Jan.: but these affections differed from each other in that something which constitutes their *proper nature,* and their hundred different shades and varieties. The same may be said of a crowd of other diseases. Thus, then, we admit figures as a means of study and verification, we reject them as a general system. The science of man is too multiple to bend itself to the rigour of their corollaries."

